# Inhibition of Oxidative Stress and ALOX12 and NF-κB Pathways Contribute to the Protective Effect of Baicalein on Carbon Tetrachloride-Induced Acute Liver Injury

**DOI:** 10.3390/antiox10060976

**Published:** 2021-06-18

**Authors:** Chongshan Dai, Hui Li, Yang Wang, Shusheng Tang, Tony Velkov, Jianzhong Shen

**Affiliations:** 1 College of Veterinary Medicine, China Agricultural University, No. 2 Yuanmingyuan West Road, Beijing 100193, China; wangyang@cau.edu.cn (Y.W.); tssfj@cau.edu.cn (S.T.); sjz@cau.edu.cn (J.S.); 2 Beijing Key Laboratory of Detection Technology for Animal-Derived Food Safety, College of Veterinary Medicine, China Agricultural University, Beijing 100193, China; 3 Beijing Key Laboratory of Diagnostic and Traceability Technologies for Food Poisoning, Beijing Center for Disease Prevention and Control, Beijing 100193, China; lihui@bjcdc.org; 4 Department of Pharmacology & Therapeutics, School of Biomedical Sciences, Faculty of Medicine, Dentistry and Health Sciences, The University of Melbourne, Parkville, VIC 3010, Australia

**Keywords:** baicalein, oxidative stress, ferroptosis, acute liver injury, ALOX12 pathway, Nrf2 pathway

## Abstract

This study investigates the protective effect of baicalein on carbon tetrachloride (CCl_4_)-induced acute liver injury and the underlying molecular mechanisms. Mice were orally administrated baicalein at 25 and 100 mg/kg/day for 7 consecutive days or ferrostatin-1 (Fer-1) at 10 mg/kg was i.p. injected in mice at 2 and 24 h prior to CCl_4_ injection or the vehicle. Our results showed that baicalein or Fer-1 supplementation significantly attenuated CCl_4_ exposure-induced elevations of serum alanine aminotransferase and aspartate aminotransferase, and malondialdehyde levels in the liver tissues and unregulated glutathione levels. Baicalein treatment inhibited the nuclear factor kappa-B (NF-κB) pathway, activated the erythroid 2-related factor 2 (Nrf2)/heme oxygenase 1 (HO-1) pathway in liver tissues, and markedly improved CCl_4_-induced apoptosis, inflammation and ferroptosis in liver tissues exposed with CCl_4_. In vitro, baicalein treatment improved CCl_4_ -induced decreases of cell viabilities and knockdown of *Nrf2* and arachidonate 12-lipoxygenase (*ALOX12*) genes partly abolished the protective effect of baicalein on CCl_4_ -induced cytotoxicity in HepG2 cells. In conclusion, our results reveal that baicalein supplementation ameliorates CCl_4_-induced acute liver injury in mice by upregulating the antioxidant defense pathways and downregulating oxidative stress, apoptosis, inflammation and ferroptosis, which involved the activation of Nrf2 pathway and the inhibition of ALOX12 and NF-κB pathways.

## 1. Introduction

Over the past several decades, liver disease has been consistently on the rise, so much so that it has become one of the leading causes of mortality and morbidity worldwide [1]. Currently, approved drugs for treating liver injury usually have many side-effects and limited efficacy [2], such that to date, safer, effective hepatoprotective drugs remain an unmet medical need.

Drugs, chemicals or alcohol-caused acute liver injury are common in most countries and serious liver injury often leads to liver failure, and even death [3]. The pathology of acute liver injury involves necrosis, infiltration of inflammatory cells and apoptosis [4,5,6,7]. Carbon tetrachloride (CCl_4_) is commonly used as the inducing agent in animal models of acute injury liver (e.g., mice, rats or rabbits), which are commonly employed to identify or screen for hepatoprotective agents [4,5,6,7]. It is well known that CCl_4_ is metabolized by the liver cytochrome P4502E1, which leads to the production of highly reactive trichloromethyl free radicals and excessive reactive oxygen species (ROS) [8]. It is well known that excessive ROS production damages intracellular macromolecules (e.g., lipids, proteins, and DNA) and leads to lipid peroxidation and oxidative stress; the two biochemical hall marks of acute liver injury [9,10,11,12]. CCl_4_ exposure induces necrosis, apoptosis and inflammatory responses in hepatocytes via several pathways, including the activation of autophagy, p53, toll-like receptor 4 (TLR4), mitogen-activated protein kinase (MAPK), and transforming growth factor-β1 (TGF-β1) pathways [13,14,15]. CCl_4_ exposure can also up-regulate the nuclear factor-kappaB (NF-κB)-mediated inflammatory response, which leads to the release and expression of tumor necrosis factor-alpha (TNF-α), high mobility group box-1 protein 1 (HMGB1), interleukin-1β (IL-1β), IL-6, and inducible nitric oxide synthase (iNOS) [13,14,15,16]. Notably, we have previously shown that the up-regulation of cellular anti-oxidant systems (superoxide dismutase [SOD], and catalase [CAT] and production of glutathione [GSH]), ameliorates CCl_4_-induced acute liver injury [13,15,17]. Recent studies revealed that ferroptosis, a form of regulated cell death, may be a key player in acute liver injury. Ferroptosis is characterized by the iron-dependent accumulation of lipid hydroperoxides to lethal cellular levels [18]. The up-regulated expression of cyclooxygenase-2 (COX-2), increased of malondialdehyde (MDA) levels and depletion of glutathione (GSH) levels are considered as the major biomarkers of ferroptosis [18]. CCl_4_ exposure-induced acute liver injury involves a similar pathology as well as increased levels of monounsaturated and polyunsaturated fatty acids (PUFA) [19]. In line with these findings, ferroptosis inhibitors (e.g., *N*-acetylcysteine (NAC) and deferoxamine (DFO) were shown to effectively improve CCl_4_-induced acute liver injury or liver fibrosis in rats [20,21].

**Figure 1:**
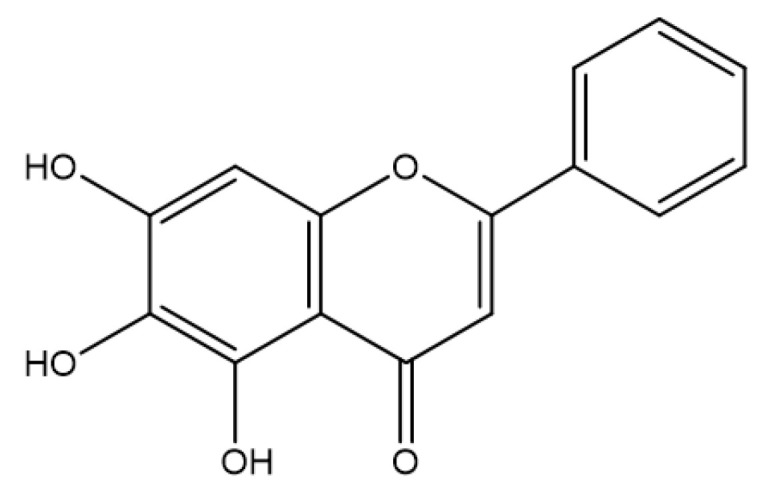
The chemical structure of baicalein.

Herbal antioxidants are attractive therapeutic alternatives for preventing or treating acute liver injury due to their higher efficiency and low incidence of adverse effects [2]. Baicalein (5,6,7-trihydroxy-2-phenylchromen-4-one; Figure 1), is a nature product from the root of *Scutellaria baicalensis*. It has many beneficial pharmacological activities, including anti-inflammatory, anti-oxidative, anti-macrobiotic and immuno-regulatory [22]. Baicalein has a time-honored place in Asian traditional medicine for treating liver, renal and cardiovascular diseases [23,24,25]. Available evidences suggests that the baicalein supplementation can attenuate cisplatin-induced hepato- and nephro-toxicity, via the attenuation of oxidative stress, apoptosis, and inflammatory responses [23,26]. A high-throughput screen from a natural product library containing 1130 chemicals showed that baicalein had highest inhibitory effects on acute liver injury induced lipopolysaccharides (LPS)/Dgalactosamine (D-gal) in vitro [27]. In the present study, we investigated the protective effects of baicalein supplementation on CCl_4_-induced acute liver injury and the underlying molecular mechanisms both in vivo and in vitro. We assume that inhibition of oxidative stress, inflammation, and arachidonate 12-lipoxygenase (ALOX12) and NF-κB pathways contributed to the protective effect of baicalein on carbon tetrachloride-acute induced liver injury.

## 2. Materials and Methods

### 2.1. Chemicals and Reagents

Baicalein (purity ≥ 98%) was purchased from Aladdin Reagent Co., Ltd. (Shanghai, China). Phenylmethylsulfonyl fluoride (PDMSF) was purchased from Sigma (St. Louis, MO, USA). CCl_4_ was purchased from Kaixing Chemical Industry Co., Ltd. (Tianjin, China). Ferrostatin-1 (Fer-1) was purchased from Cayman Chemicals (Ann Arbor, MI, USA). Erastin was purchased from Selleck Chemicals (Shanghai, China). All other reagents were of the highest analytical reagent grade available.

### 2.2. Animals, Treatments and Experimental Design

The Institutional Animal Care and Use Committee at the China Agricultural University approved all animal experiments in this study. Male C57 BL/6 mice (8 weeks old, 20–22 g) were purchased from Vital River Animal Technology Co., Ltd. (Beijing, China). An acclimation period of one week for all animals was performed. Mice were housed in a room maintained under the following conditions: at a temperature of 22 ± 3 °C and relative humidity of 55 ± 5% and a 12-h light-dark cycle. During the experiments, all mice had free access to food and water. In all animal experiments, mice were euthanized by intraperitoneal injection of an overdose of sodium pentobarbital (80 mg/kg) (Sigma, St. Louis, MO, USA).

#### 2.2.1. Gene and Protein Expression in the Liver Tissues of Mice Exposed CCl_4_

To investigate the gene and protein expressions in the liver tissues after CCl_4_ exposure, eight mice were intraperitoneally (i.p) injected with 0.3% CCl_4_ (10 mL/kg, dissolved in olive oil). The mice in the control group were i.p. injected with an equal volume of the olive oil vehicle. At 6 and 24 h, mice were sacrificed (4 mice were in each time point) and liver tissues were collected. Key genes and protein as the biomarkers of apoptosis (*Bax*, *GADD45a*, *MAPK7*, and *p21*), inflammation (*NF-κB*, *TNF-a*, *IL1β*, *IL-6*, and *iNOS*), and ferroptosis (*GPX1*, *GPX4*, *ALOX12*, *SCD1*, and *COX-2*) pathways were selected and examined.

#### 2.2.2. Effect of Baicalein Supplementation on CCl_4_-Induced Acute Liver Injury

To investigate the protective effect of baicalein supplementation on CCl_4_-induced acute liver injury, sixty mice were randomly divided into six groups (10 mice were in each group): (1) untreated control, (2) baicalein 25 mg/kg/day (designated Bai 25), baicalein 100 mg/kg/day (designated Bai 100), (4) CCl_4_ only, (designated CCl_4_) (5) CCl_4_ plus baicalein 25 mg/kg/day (designated CCl_4_ + Bai 25) and (6) CCl_4_ plus baicalein 100 mg/kg/day (designated CCl_4_ + Bai 100). Baicalein was dissolved in 0.5% sodium carboxymethyl cellulose (CMC-Na) and orally administrated to mice at 25 mg/kg or 100 mg/kg per day for 7 consecutive days, which was accorded to the previous published studies [28,29]; the mice in the control group were treated with an equal volume of 0.5% CMC-Na. After final administration, the mice in the CCl_4_ + Bai 25; CCl_4_ + Bai 100; and CCl_4_ only groups were injected intraperitoneally (i.p.) with 0.3% CCl_4_ (10 mL/kg, dissolved in olive oil). The control and baicalein alone groups (i.e., Bai 25 and Bai 100) were i.p. administered with an equal volume of the olive oil vehicle only. Following 24 h after CCl_4_ challenge, mice were sacrificed; blood was collected and serum alanine aminotransferase (ALT) and aspartate aminotransferase (AST) were evaluated. The liver samples were collected for histopathological and biochemical assessments including the biomarkers of oxidative stress, apoptosis staining, and gene expression.

#### 2.2.3. Ferroptosis in Liver Acute Injury

To investigate the role of ferroptosis in CCl_4_ -induced acute liver injury, mice were co-administered Fer-1, a potent and selective inhibitor of ferroptosis [30]. Twenty-four mice were randomly divided into 4 groups (6 mice in each group): (1) control, (2) Fer-1, (3) CCl_4_, and (4) CCl_4_ plus Fer-1 (CCl_4_ + Fer-1). The dose of Fer-1 is accorded to the Yamada et al. study with minor revision [31]. Fer-1 were i.p. injected at 10 mg/kg to mice for 2 times before CCl_4_ exposure, i.e., at 24 h and 2 h prior to CCl_4_ exposure. At 24 h post CCl_4_ challenge, blood and liver samples were harvested. The levels of AST and ALT, and histopathological changes, were evaluated.

### 2.3. In Vivo Animal Experiment and Measurement

#### 2.3.1. Levels of ALT and AST

Blood samples from mice were centrifuged at 3000× *g* for 10 min for biochemical analysis, according to a previously published paper with minor revision [32]. Serum alanine aminotransferase (ALT) and aspartate aminotransferase (AST) activities were measured using an automatic analyzer (Hitachi 7080, Hitachi High-Technologies Corporation, Tokyo, Japan).

#### 2.3.2. Histopathological Examination of Liver Tissues

Liver tissues from four mice were isolated and fixed in 10% neutral buffered formalin for at least 48 h, then sectioned and histopathological analysis were performed as described below [15]. In brief, the liver slices were cut into 4 μm-thick sections and stained with hematoxylin-eosin. A semi-quantitative score (SQS) system was used to analyze the histopathological damage: Grade 0 showed no marked pathological change. Grade 1 showed the presence of hepatocyte degeneration with small rare of foci of necrosis. Grade 2 showed the small area of mild centrilobular necrosis around the central vein. Grade 3 showed the area of mild centrilobular necrosis that is severer than Grade 2. Grade 4 showed the centrilobular necrosis that is severer than Grade 3.

#### 2.3.3. Measurement for Antioxidant Markers

Approximately 100 mg of liver tissue was homogenized in 1 mL of cold Tris buffer to prepare a 10% tissue homogenate, according to previous published paper with minor revision [32]. Then, the homogenates were centrifuged at 3000× *g* for 15 min at 4 °C. The supernatant was collected for the measurement of the levels of MDA, nitric oxide (NO) and reduced glutathione (shown as GSH) and activities of iNOS (Beyotime, Haimen, China), CAT, and SOD using commercial kits as per the manufacturer’s instructions (Nanjing Jiancheng Institute of Biological Engineering, Nanjing, China), respectively.

#### 2.3.4. Measurement of *IL-1β*, *TNF-α*, and *IL-6* Levels in Liver Tissues

The levels of *TNF-a*, *IL-1β* and *IL-6* in the liver tissues were determined by using *TNF-a*, *IL-1β* and *IL-6* ELISA kits according to the manufacturer’s instructions (R&D Systems, Minneapolis, MN, USA).

#### 2.3.5. Measurements of Caspase-9 and -3 Activities

Approximately 10 mg of liver tissue was lysed using the commercially supplied cold lysis buffer (about 0.5 mL) for 15 min. The lysates were centrifuged at 12,000× *g* for 10 min at 4 °C and the supernatants were collected. The activities of caspase-3 and caspase-9 were measured using commercial caspase-3 and caspase-9 kits according to the manufacturer’s instructions (Beyotime, Haimen, China). The activities were normalized to the value for the untreated control samples. 

#### 2.3.6. Measurements of Apoptosis in the Liver Tissues

The apoptosis rates in the liver slices were measured using a deoxynucleotidyl transferase-mediated dUTP nick-end labeling (TUNEL) kits (Vazyme Biotech Co., Ltd., Nanjing, China) and 4′-6-diamidino-2-phenylindole (DAPI) was used to label the cell nuclei.

#### 2.3.7. Quantitative Reverse-Transcription (qRT) PCR Examination

Gene expression analysis were performed by qRT-PCR. In brief, total RNA was isolated using the TRIzol extraction method (Invitrogen Inc, Carlsbad, CA, USA). The quality of isolated RNA was evaluated by the optical density at 260/280 nm (all values are between 1.9~2.1). Approximately 500 ng of total RNA was subjected to reverse transcription to produce cDNA by using the Prime Script RT-PCR kit (Takara, Dalian, China). The detail primer information is documented in Supplementary Table S1. β-actin was used as an internal control, and 2^−ΔΔCt^ method was used to calculate the fold change of gene expression.

#### 2.3.8. Western Blotting

Mouse liver tissue of 10–20 mg was homogenized using a Dounce homogenizer in 500 µL ice-cold lysis buffer (100 mM Tris-HCl, 2% (*w*/*v*) sodium dodecyl sulfate (SDS), 10% (*v*/*v*) glycerol, pH 7.4) with a protease inhibitor cocktail (1 μg/mL aprotinin, 1 μg/mL leupeptin, 1 μg/mL pepstatin A and 1 mM PMSF). Tissue lysate samples were centrifuged at 12,000× *g* for 15 min at 4 °C, and the protein concentration was measured using a bicinchonininc acid (BCA) protein assay kit. Equal amounts of protein from each sample were resolved by sodium dodecyl sulfate-polyacrylamide gel electrophoresis (SDS-PAGE) and transferred to nitrocellulose membranes (Bio-Rad, Hemel Hempstead, UK). The following primary antibodies were used to probe the membranes: primary rabbit antibodies against p21 (1:1000), COX2 (1:1000), Nrf2 (1:1000), and HO-1 (1:1000) (ProteinTech Group, Inc., Chicago, IL, USA), mouse monoclonal antibody against ALOX12 (1:1000) (Abcam, Cabridge, MA, USA) and β-actin (1:1000) (Santa Cruz Biotechnology, Dallas, TX, USA). Membranes were incubated overnight with the primary antibodies, followed by developing with secondary antibodies (1:5000) for 1 h at room temperature. The results were quantified by densitometry using Image J software, and normalized relative to the β-actin bands.

### 2.4. Cell Culture, Treatment and Measument

#### 2.4.1. Cell Culture

The human HepG2 cell line was purchased from the Cell Bank of the Chinese Academy of Sciences (Shanghai, China). HepG2 cells were cultured in Dulbecco’s modified Eagle’s medium (DMEM) medium (Life Technologies Corporation, Grand Island, NY, USA) containing 10% (*v*/*v*) heat-inactivated fetal bovine serum (FBS), 100 units/mL penicillin, 110 mg/L sodium pyruvate, and 100 μg/mL streptomycin (Beyotime, Haimen, China) and maintained in a humidified atmosphere of 95% air and 5% CO_2_ at 37  °C.

#### 2.4.2. Gene Knockdown by siRNA

HepG2 cells were transfected with siRNA oligos (Sigma, St. Louis, MO, USA) that targeted *Nrf2* and *ALOX12* using Lipofectamine RNAiMAX (ThermoFisher, Cabridge, MA, USA) in Opti-MEM™ for 6 h. The medium was then replaced by fresh DMEM for additional 24 h. For each targeted gene, two commercial siRNA oligos were employed: *Nrf2*: SiRNA#1, SASI_Hs01_00182393; SiRNA#2, SASI_Hs02_00341015; *ALOX12*: SiRNA#1, SASI_Hs02_00303100; SiRNA#2, SASI_Hs02_00303101. Mission siRNA universal negative control #1 (Sigma, St. Louis, MO, USA) was used as a negative control. Various treatments were performed at 24 h after knockdown. To confirm the effectiveness of siRNA, the expressions in the levels of mRNA and protein were measured by using qRT-PCR and Western blotting.

#### 2.4.3. Measurement of Cell Viability

HepG2 cells were seeded in 96-well plate (1 × 10^5^ cells/well) for 12 h, then cells were pretreated with baicalein at 10 and 20 μM for 2 h, or knockdown with *Nrf2* or *ALOX12* genes, respectively, then treated with 10 μM erastin or 0.4% CCl_4_ for additional 24 h. After treatment, a fresh medium containing 10 μL CCK-8 solution was added into each well of the plate and incubated for a further 1 h. Then, CCK-8 absorbance was read using a microplate reader at 450 nm (Tecan Trading AG, Zürich, Switzerland).

#### 2.4.4. Levels of MDA in HepG2 Cells

To examine the protective effect of baicalein treatment on CCl_4_ induced cell death, HepG2 cells were pre-treated baicalein at 10 and 20 μM for 2 h, then treated with 0.4% CCl_4_ (*v*/*v*) for additional 24 h. The levels of MDA were also measured in the cell samples as detailed above.

### 2.5. Statistical Analysis

All data are expressed as mean ± standard deviation (SD) unless specifically stated. All figures were drawn by using Graph Pad Prism 8.2 (Graph Pad Software, Inc. San Diego, CA, USA) and statistical differences were performed by using one-way analysis of variance from Graph Pad Prism 8.2. A Tukey’s multiple comparisons test was used to compare any two means when the variance was homogeneous, otherwise, Dunnett’s T3 test was used. A *p* value < 0.05 was considered as statistical significance.

## 3. Results

### 3.1. CCl_4_-Induced Acute Liver Damage Involves Upregulation of Genes Involved in Oxidative Stress, Inflammation, Apoptosis and Ferroptosis Pathways

**Figure 2:**
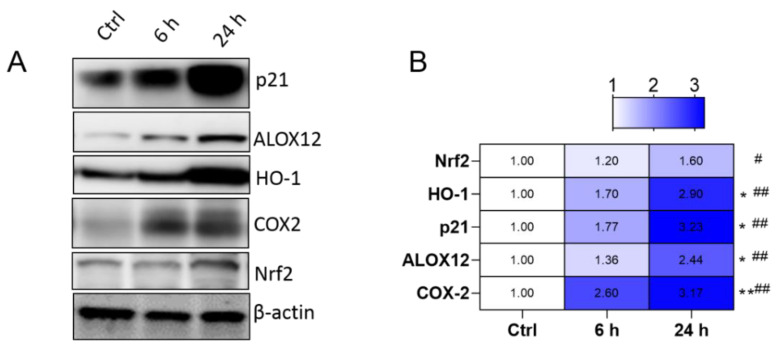
Protein expression levels involved ferroptosis were detected in the liver tissues of mice challenged with CCl_4_. (**A**) Protein expression levels of *ALOX12*, *HO-1*, *COX-2*, *p21* and *Nrf2* at 6 and 24 h identified by Western blotting analysis of liver tissues of mice treated with CCl_4_; the quantitative analysis is showed in (**B**) (*n* = 4). 6 h vs. ctrl, * *p*  <  0.05, ** *p*  <  0.01; 24 h vs. ctrl, ^#^ *p*  <  0.05, ^##^ *p*  <  0.01. Ctrl: control.

The liver tissue gene expression levels at 24 h in mice exposed with CCl_4_ are shown in Supplementary Figure S1. Notably, the mRNA expression of genes enriched in the Nrf2 pathway (*Nrf2* and *HO-1* mRNAs increased to 1.73- and 4.68-fold, respectively), apoptosis (*Bax*, *GADD45a*, *MAPK7*, and *p21* mRNAs increased to 3.09-, 3.56-, 3.32-, 3.12-fold, respectively), inflammation (*NF-κB*, *TNF-a*, *IL1β*, *IL-6*, and *iNOS* mRNAs increased to 4.60-, 6.50-, 7.54-, 5.32-, and 4.53-fold, respectively) and ferroptosis pathways (*GPX1*, *GPX4*, *ALOX12*, *SCD1*, and *COX-2* mRNAs increased to 2.83-,3.55-, 3.45-, 3.19-, and 4.27-fold, respectively) were significantly up-regulated, compared to that in the control group. Furthermore, we also identified from the Western protein expression results that, at 24 h, CCl_4_ exposure significantly unregulated the levels of *Nrf2*, *HO-1*, *ALOX12*, *COX-2* and *p21* protein expression, compared to that in the control groups (Figure 2A,B).

### 3.2. Baicalein and Fer-1 Supplementation Ameliorates CCl_4_-Induced Acute Liver Injury

**Figure 3:**
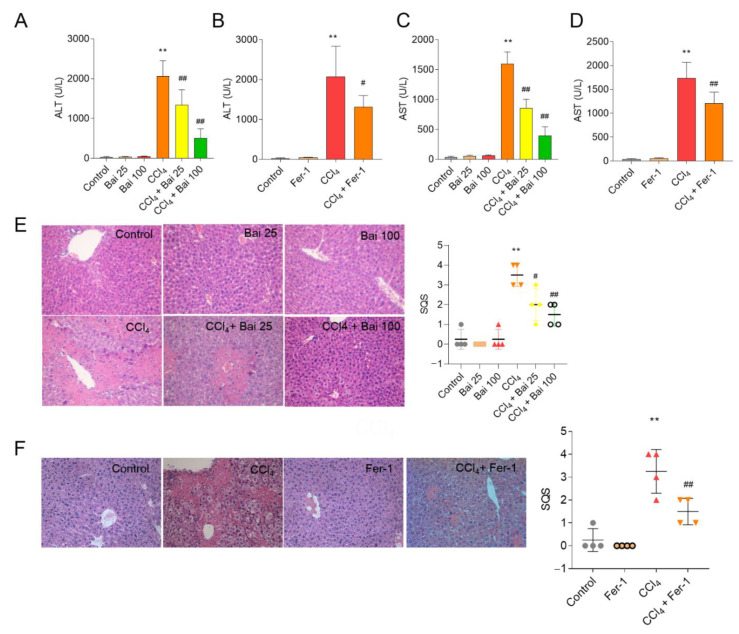
Effects of baicalein and Fer-1 supplementation on CCl_4_-induced liver dysfunction and histopathology. Levels of serum (**A**,**B**) ALT and (**C**,**D**) AST (*n* = 6). (**E**,**F**) Representative histopathological images of hematoxylin and eosin (H&E) stained liver sections and their semi-quantitative scoring (SQS (*n* = 4)). Magnification, ×20. All results are presented as mean ± SD. ** *p* < 0.01, compared to the untreated control group, respectively; ^#^ *p* < 0.05 or ^##^ *p* < 0.01 compared to the CCl_4_ only group. Bai, baicalein. Fer-1, ferrostatin-1.

The hepatoprotective effects of baicalein and Fer-1 supplementation on CCl_4_-induced acute liver injury in mice were accessed through the measurement of AST and ALT serum levels and histopathological examination of liver sections (Figure 3). As shown in Figure 3A–D, CCl_4_ exposure significantly increased the serum AST and ALT activities (both *p* < 0.01) compared to the untreated control group. Baicalein supplementation at doses of 25 and 100 mg/kg/day for 7 days (i.e., CCl_4_ + Bai 25 and CCl_4_ + Bai 100 groups) or Fer-1 administration at 10 mg/kg markedly decreased the levels of serum AST and ALT (Figure 3A–D), compared to the CCl_4_-only group. Consistently, marked histopathological liver injury (SQS increased to 3.5) was detected at 24 h after CCl_4_ exposure, wherein large areas of cellular necrosis and numbers inflammatory cell infiltration were detected (Figure 3E,F). Baicalein or Fer-1 supplementation also significantly improved CCl_4_ induced pathology damage; the histological scores significantly decreased to 2.0 (*p* < 0.05), 1.5 (*p* < 0.01) and 1.5 (*p* < 0.01) in the CCl4+ Bai 25, CCl_4_ + Bai 100 groups, or CCl_4_ + Fer-1 groups, respectively (Figure 3E,F).

### 3.3. Baicalein and Fer-1 Supplementation Ameliorates CCl_4_ -Induced Ferroptosis

**Figure 4:**
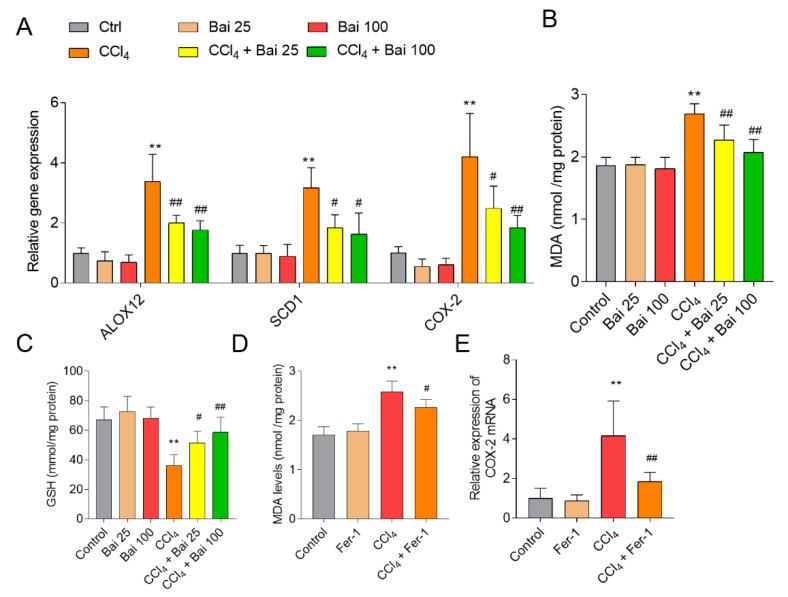
Effects of baicalein and Fer-1 supplementation on the expression of *COX-2*, lipid peroxidation and GSH in the liver tissues of mice challenged with CCl_4_. (**A**) Effect of baicalein supplementation on expression of *ALOX12*, *SCD1*, and *COX-2* mRNAs in liver tissues. (**B**) Effect of baicalein supplementation on the levels of MDA in liver tissues. (**C**) Effect of baicalein supplementation on the levels of reduced GSH (shown as GSH) in liver tissues. (**D**) Effect of Fer-1 supplementation on the levels of MDA in liver tissues. (**E**) Effect of Fer-1 supplementation on the expression of COX-2 mRNA in liver tissues. All data are presented as mean ± SD (*n* = 6). ** *p* < 0.01, compared to the untreated control group, respectively; ^#^ *p* < 0.05 or ^##^ *p* < 0.01 compared to the CCl_4_-only group. MDA: malondialdehyde; GSH: glutathione; Bai: baicalein.

Baicalein supplementation markedly inhibited the expression of markers of ferroptosis including *SCD-1*, *ALOX12* and *COX-2* mRNAs in the liver tissues of mice, compared to that in the CCl_4_ only group (Figure 4A). Baicalein supplementation also significantly improved CCl_4_-induced lipid peroxidation (CCl_4_ only group MDA = 2.69 mmol/mg; CCl_4_ + Bai 25 MDA = 2.27 mmol/mg; CCl_4_ + Bai 100 MDA = 2.07 mmol/mg; both *p* < 0.01) and recovered GSH levels (Figure 4B,C). The MDA levels (*p* < 0.01) and the expressions of *COX-2* mRNA were also reduced following Fer-1 administration, compared to the CCl_4_-only group (Figure 4D,E).

### 3.4. Baicalein Supplementation Activates Liver Antioxidant Defense Pathways

**Figure 5:**
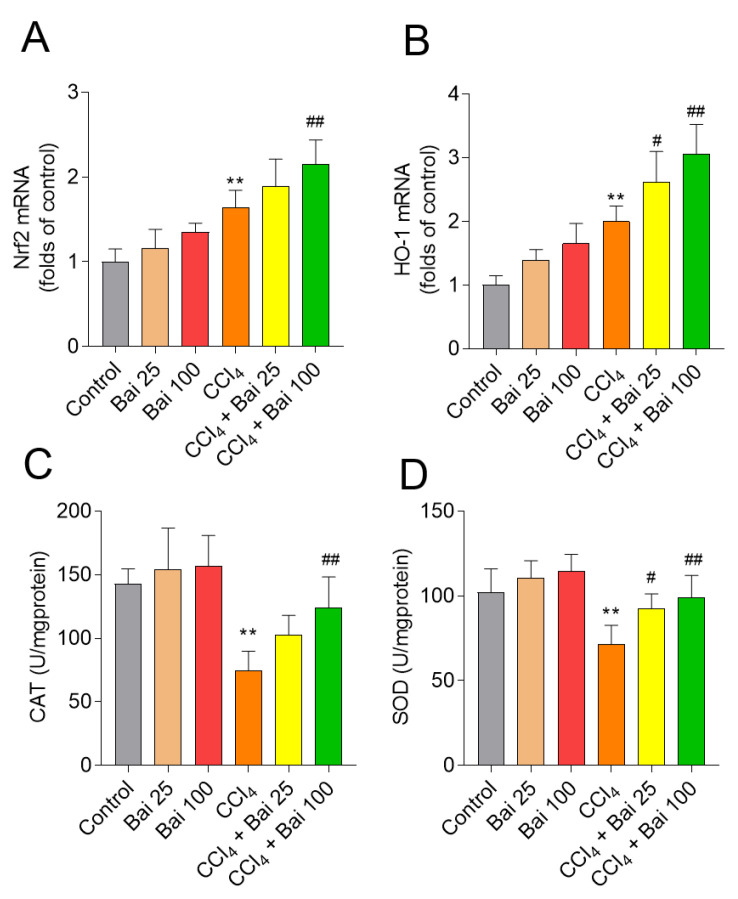
Effects of baicalein supplementation on the antioxidant defense pathways in liver tissues of mice challenged with CCl_4_. (**A**) *Nrf2* mRNA; (**B**) *HO-1* mRNA; (**C**) CAT activity; and (**D**) SOD activity. Data are presented as mean  ±  SD (*n* = 6). ** *p*  <  0.01, compared to the control group; ^#^ *p* < 0.05 or ^##^ *p*  < 0.01 compared to the CCl_4_ model group. Bai: baicalein.

As shown in Figure 5, at 24 h, CCl_4_ exposure per se up-regulated the expression of *Nrf2* and *HO-1* mRNAs compared to that in the control group (1.6- and 2.0-fold, respectively; both *p* < 0.01) (Figure 5A,B). Baicalein supplementation, particularly at 100 mg/kg/day, further up-regulated the expressions of *Nrf2* and *HO-1* mRNAs (all *p* < 0.01), compared to that in the CCl_4_ only group. Moreover, baicalein supplementation at the doses of 25 and 100 mg/kg/day for 7 consecutive days, significantly up-regulated the activities of antioxidant enzymes SOD and CAT in the liver tissues, compared to the CCl_4_-only group (Figure 5C,D).

### 3.5. Baicalein Supplementation Attenuates CCl_4_-Induced Caspase Activation and Apoptosis in the Liver Tissues

**Figure 6:**
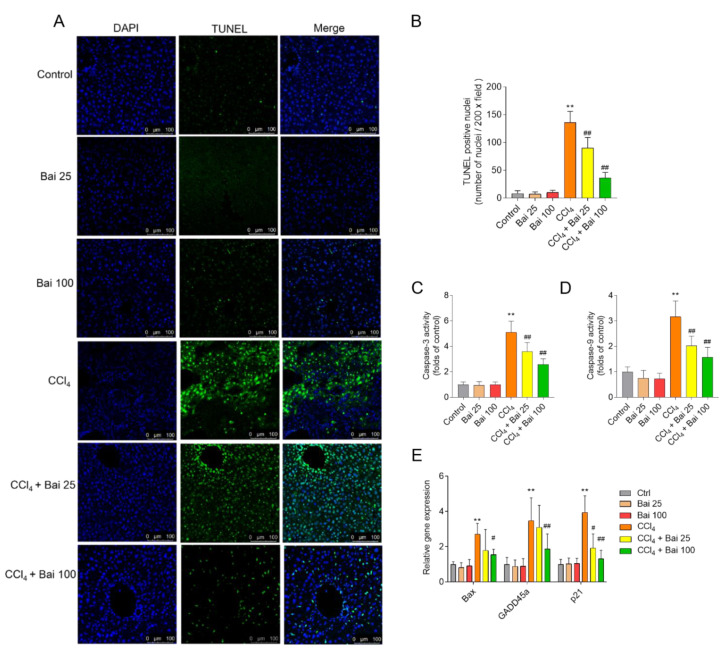
Effects of baicalein supplementation on apoptotic pathway in the liver tissues of mice challenged with CCl_4_. (**A**) Representative TUNEL-stained sections showing apoptosis in the liver tissue of mice. (**B**) Quantitative analysis of TUNEL positive rates (*n*  =  4). (**C**,**D**) Activities of (**C**) caspases-3 and -9 (**D**) (*n* = 6). (**E**) The relative expression of *Bax*, *GADD45a* and *p21* mRNAs in the liver tissues (*n*  =  5). ** *p* < 0.01, compared to the control group; ^#^ *p* < 0.05 or ^##^ *p* < 0.01, compared to the CCl_4_ only group. Bar  =  100 μm. Bai: baicalein.

TUNEL staining showed that baicalein supplemented mice displayed attenuated cellular apoptotic rates in their liver tissues (Figure 6A,B). Baicalein supplementation at the doses of 25 and 100 mg/kg/day for consecutive 7 days significantly decreased the activity of caspase-3 to 3.5- and 2.6- fold, respectively; and decreased the caspase-9 to 2.0- and 1.6- fold, respectively (Figure 6C,D). In addition, baicalein supplementation at 100 mg/kg/day also inhibited the expression of *Bax*, *GADD45a*, and *p21* mRNAs, all of which were significantly elevated following CCl_4_ exposure (Figure 6E). There were no marked changes in the activities of caspase-9 and -3, the rate of cellular apoptosis and mRNA expression of *Bax*, *GADD45a*, and *p21* in the Bai 25 and Bai 100 groups, compared to the untreated control group.

### 3.6. Baicalein Supplementation Ameliorates the NF-κB Mediated Inflammatory Response

**Figure 7:**
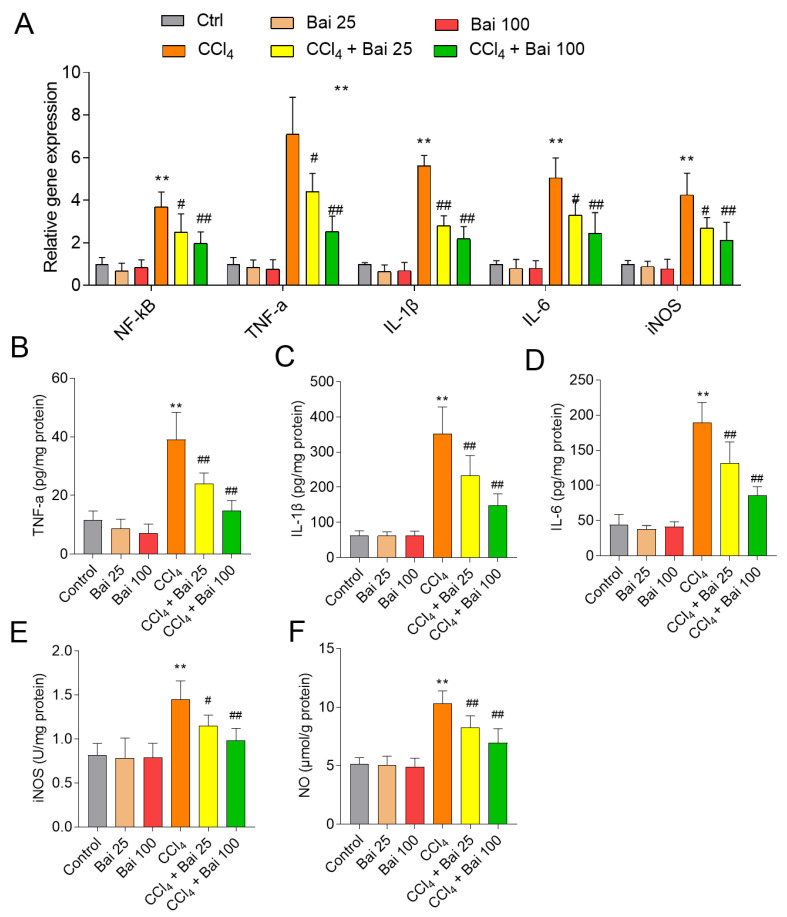
Effects of baicalein supplementation on CCl_4_ -induced inflammation in the liver tissues of mice. (**A**) The relative expression levels of *NF-κB*, *TNF-a*, *IL-1β*, *IL-6*, and *iNOS* mRNAs; (**B**) levels of *IL-1β* protein; (**C**) levels of *IL-6* protein; (**D**) levels of *TNF-a* protein; (**E**) levels of *iNOS* protein; (**F**) levels of NO. All data are presented as mean  ±  SD (*n* = 6). ** *p*  <  0.01, compared to the control group; ^#^ *p*  < 0.05 or ^##^ *p*  < 0.01, compared to the CCl_4_ only group. Bai: baicalein.

The liver tissues of mice treated with CCl_4_ alone displayed significantly elevated levels of *NF-kB*, *IL-1β*, *IL-6*, *TNF-α*, and *iNOS* mRNAs (Figure 7A), compared to untreated mice. Baicalein supplementation at the doses of 25 and 100 mg/kg/day markedly attenuated CCl_4_ exposure-induced the increases in the levels of *IL-1β*, *IL-6* and *TNF-α* in the liver tissues (Figure 7B–D). Baicalein supplementation also decreased CCl_4_ induced increases of *iNOS* and *NO* in the liver tissues (Figure 7E,F, respectively). Baicalein supplementation at 25 and 100 mg/kg/day alone did not perturb the level of any of the aforementioned inflammatory markers.

### 3.7. Baicalein Attenuates CCl_4_-Induced Cell Death in HepG2 Cells

**Figure 8:**
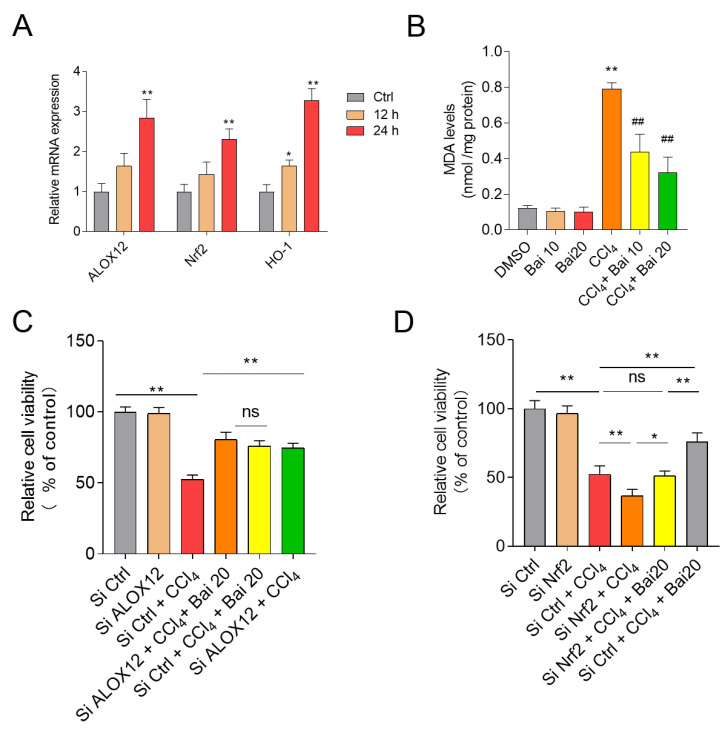
Effect of *ALOX12* and *Nrf2* genes knockdown on the protective effect of baicalein in HepG2 cells. (**A**) The expression of *ALOX12*, *Nrf2*, and *HO-1* mRNAs were examined after 0.4% CCl_4_ treatment for 12 h and 24 h in HepG2 cells. Data are presented as mean  ±  SD (*n* = 3). * *p*  <  0.05, ** *p*  <  0.01, compared to the control group. (**B**) Effect of baicalein supplementation on levels of MDA in HepG2 cells. Knockdown of *ALOX12* (**C**) or *Nrf2* (**D**) on the protective effect of baicalein on CCl_4_ (0.4%) treatment (for 24 h) -induced cell death. SiRNA#1s targeting *ALOX12* and *Nrf2* gene were used here, respectively. All data are presented as mean  ±  SD (*n* = 3). * *p*  <  0.05, ** *p*  <  0.01, compared to the SiCtrl group. ^##^ *p*  <  0.01, compared to the CCl_4_ alone group. Ctrl: control. ns: no significance.

As shown in Figure 8A, CCl_4_ exposure for 24 h significantly unregulated the expression of ALOX12, *Nrf2* and *HO-1* mRNAs in HepG2 cells. Baicalein supplementation at 10 μM and 20 significantly inhibited CCl_4_- or erastin-induced cell death (Supplementary Figure S2) and significantly inhibited the increase in MDA levels in HepG2 cells (Figure 8B). Knockdown of *ALOX12* and *Nrf2* genes by SiRNAs were confirmed by monitoring mRNA and protein expression (Supplementary Figure S3). Knockdown of *ALOX12* gene significantly inhibited erastin- or CCl_4_-induced cell death in HepG2 cells (Figure 8C and Supplementary Figure S4A) and abolished the protective effect of baicalein. In contrast, knockdown of *Nrf2* gene significantly promoted erastin- or CCl_4_-induced cell death and partly abolished the protective effect of baicalein (Figure 8D and Supplementary Figure S4B).

## 4. Discussion

Liver disease is a global public health burden that causes one million deaths annually [1]. Currently available hepato-protective drugs are limited in efficacy or elicit many adverse reactions [33]. These factors highlight the urgent need for novel hepatoprotective agents with low or no adverse effects. Natural products have been humankind’s most fruitful source of drugs [34]. In line with previous reports, the present study showed that CCl_4_ exposure significantly up-regulated AST and ALT levels [35]. Histological assessment of the liver tissues of mice treated with CCl_4_ per se revealed hepatotoxicosis seen as fragmented, condensed and lost nuclei. Supplementation with baicalein markedly attenuated CCl_4_-induced acute liver injury in mice via the inhibition of oxidative stress, ferroptosis, caspase activation, apoptosis, and NF-κB-mediated inflammation (Figure 2, Figure 3, Figure 4, Figure 5, Figure 6 and Figure 7).

It is well known that CCl_4_ induced hepatoxicity partly depends on the production of excessive ROS, which in turn causes damage to cellular lipids, DNA, and proteins [13,14,15]. Lipid peroxidation is one of the principal hallmarks of CCl_4_-induced liver injury [36]. Lipid peroxides react with metals such as iron, resulting in many reactive carbonyls (e.g., 4-hydroxynonenal and MDA). These reactive carbonyls become strongly conjugated to GSH and in turn deplete cellular GSH levels. In the current study, we detected markedly depleted GSH levels, increased MDA (a biomarker of lipid peroxidation) in the liver tissues of mice at 24 h post CCl_4_ exposure [37], which were all markedly improved by baicalein supplementation. The anti-oxidant properties of baicalein have been associated with its ability to ameliorate cisplatin, colistin and hypoxia-reoxygenation-induced nephrotoxicity and doxorubicin-induced cardiotoxicity in mice [23,28,38,39]. The strong anti-oxidant property of baicalein is conferred by its three 5,6,7 position OH-groups that play a critical role in scavenging ROS [26,40]. In the present study, baicalein supplementation markedly inhibited CCl_4_-induced increases in the levels of NO and *iNOS* activities (Figure 7), two biomarkers of nitrative stress [41]. NO can react with superoxide leading to formation of highly reactive peroxynitrite, causing DNA damage and apoptotic cell death [42,43]. Collectively, our findings indicate that baicalein can protective CCl_4_-induced acute liver injury by inhibiting nitrative stress.

Recently, Choi et al., reported that baicalein is an activator of nuclear factor-erythroid 2-related factor 2 (*Nrf2*), a critical redox-sensitive transcription factor in the cellular anti-oxidant response [44,45,46,47,48]. The activation of *Nrf2* could attenuate cellular oxidative damage by activating genes that encode phase II detoxifying enzymes and antioxidant enzymes, such as *CAT*, *SOD*, and *HO-1* [41]. CCl_4_ exposure is known to cripple the *Nrf2* antioxidant defense system and exacerbate oxidative damage in the liver [14,36]. Consistently, we found that CCl_4_ exposure significantly decreased the activities of *SOD* and *CAT* in the liver tissues of mice, whereas baicalein supplementation markedly up-regulated the activities of *SOD* and *CAT*; and activated *Nrf2* expression and its downstream gene *HO-1*. Silencing *Nrf2* at the genetic level has been shown to diminish the protective effect of baicalein on H_2_O_2_-induced oxidative damage in HEI193 Schwann and NG108-15 cells [47,48,49]. Similarly, baicalein supplementation has been shown to markedly improve colistin-induced nephrotoxicity in mice via the activation of *Nrf2/HO-1* pathway and inhibition of *NF-κB* mediated inflammatory response [28]. Coincidently, knockdown of *Nrf2* inhibited erastin-induced ferroptotic cell death in human HepG2 cells (Figure 8), indicating that Nrf2 potentially plays an important role in ferroptosis [50]. In the present study, CCl_4_ exposure significantly increased the expression of *Nrf2* mRNA and protein levels and the expression of its downstream gene *HO-1* in the liver tissues of mice (Figure 5). Baicaien supplementation counteracted these negative perturbations and significantly unregulated the expression of *Nrf2* and *HO-1* mRNA in the liver tissues (Figure 5). Consistently, knockdown of *Nrf2* by RNAi silencing promoted CCl_4_-induced cell death in HepG2 cells and partly abolished the protective effect of baicalein (Figure 8). Taken together, our results suggested that baicalein protects against CCl_4_ induced acute liver injury via activation of the *Nrf2/HO-1* anti-oxidant pathway.

Our present results showed that baicalein supplementation markedly attenuated CCl_4_ -induced activation of caspases-9 and -3 and cellular apoptosis; and inhibited *p21*, *GADD45a* and *Bax* expression (Figure 6). Caspase-9 is an important biomarker in mitochondrial-dependent apoptosis [45]. Mitochondria are particularly susceptible to ROS [51]; coincidently, CCl_4_ exposure has been associated with marked mitochondrial dysfunction, resulting in cytochrome C release which triggers the mitochondrial apoptotic death pathway [15,52]. In previous studies, baicalein supplementation was shown to markedly inhibit colistin-induced activation of caspases-9 and -3 in the kidney tissues of mice or inhibit CCl_4_ exposure-induced mitochondrial apoptotic cell death in liver tissues of mice [28,53]. Similarly, Jeong and colleagues reported that baicalein treatment (100 μM) markedly inhibited H_2_O_2_-induced oxidative stress, caspases activation, and apoptosis in HEI193 Schwann cells [54]. Taken together, our results indicated that baicalein supplementation ameliorates toxin-induced oxidative stress in the liver, thereby preventing mitochondrial dysfunction and apoptotic cell death.

The inflammatory response is one of the main events associated with the pathological progress of CCl_4_ -induced acute liver injury [4,5,6,10,12,55,56,57,58]. NF-κB is a critical transcriptional mediator of pro-inflammatory cytokine expression, such as IL-1β, IL-6, TNF-α, and *COX-2* [59]. It has been reported that baicalein treatment of macrophages down-regulated lipopolysaccharide-induced production of TNF-α, IL-6 and iNOS [60]. Wu et al. demonstrated that oral administration of baicalein can improve D-galactosamine or lipopolysaccharide or CCl_4_-induced acute liver failure and liver fibrosis by blocking the recruitment of *NF-κB* signaling [42,61,62]. Consistently, our results showed that baicalein supplementation markedly inhibited the production of IL-1β, TNF-α, and IL-6 levels, and the expression of *NF-κB* mRNA in the liver tissues of mice exposed to CCl_4_ (Figure 7). Notably, *Nrf2* activation can lead to the inhibition of oxidative stress driven by *NF-κB* nuclear translocation mediated inflammatory response via *HO-1* end-products (i.e., bilirubin) [59]. Therefore, the activation of Nrf2/HO-1 pathway caused by baicalein may partly contribute to the inhibition of NF-kB-mediated inflammatory response, nonetheless, the precise molecular mechanism requires further investigation.

Ferroptosis is a type of regulated cell death characterized by iron-dependent accumulation of lipid hydroperoxides [18,30]. Ferroptosis is triggered when intracellular glutathione peroxidase 4 (GPX4) is inhibited directly; or indirectly by a decrease in cellular GSH levels [18,30]. CCl_4_-induced acute liver damage displays some hall mark features of ferroptosis, including increased lipid peroxidation and expression of *COX-2*, *p53*, *SAT1* and GSH depletion [63,64]; and is inhibited by prior- or co-administration of the ferroptosis inhibitors desferrioxamine, GSH, or NAC [19,20,21]. A recent study demonstrated that ferroptosis participated in acetaminophen-induced acute liver injury in a mouse model [31]. A high-throughput natural product library screen for ferroptosis inhibitors identified baicalein as a potential hit [65]. COX-2 is considered as a biomarker of ferroptotic cell death in murine models of acute liver injury, acute renal failure and cardiomyopathy [31,66,67]. In the present study, we showed that baicalein significantly ameliorated CCl_4_-induced lipid peroxidation, GSH depletion and *COX-2* mRNA expression. Similarly, Fer-1, an inhibitor of ferroptosis, also significantly inhibited CCl_4_-induced acute liver injury. Fer-1 also significantly inhibited the expression of *COX-2* mRNA and the levels of MDA in the liver tissue of mice. In addition, baicalein supplementation inhibited ferroptosis in HepG2 cells following erastin treatment. Taken together, these findings suggest that baicalein supplementation can inhibit CCl_4_-induced ferroptosis that partly contributes to its protective effect on CCl_4_-induced acute liver injury in mice.

Baicalein is a known inhibitor of 12/15-lipoxygenases (12/15-LOXs), two seminal enzymes involved in the metabolism of polyunsaturated fatty acids [68]. In the present study, we demonstrated that CCl_4_ exposure increased the expression of stearoyl-CoA desaturase-1 (*SCD1*) and *ALOX12*, which are the positive re-enforcers in the process of ferroptotic cell death [69]. Baiclaein has been shown to inhibit ischemia-reperfusion-induced cardiac damage in rats via the inhibition of *ALOX12* activity [70] wherein ferroptosis is an underlying mechanism [67]. In the current study, baicalein supplementation markedly inhibited the expression of *SCD1* and *ALOX12* in the liver tissues of mice. Furthermore, our present study showed that knockdown of *ALOX12* significantly inhibited ferroptosis following erastin- and CCl_4_-induced HepG2 cell death. Furthermore, silencing *ALOX12* significantly abolished the protective effect of baicalein (Figure 8). This would suggest that baicalein mediated inhibition of CCl_4_-induced ferroptosis is partly dependent on the inhibition of *ALOX12* activity. Previous studies also showed that inhibition of *ALOX12* gene expression also contributed to inhibition of inflammatory response [71]. Whether the inhibition of *ALOX12* gene expression contributes significantly to the inhibitory effect of baicalein on CCl_4_–induced inflammatory still requires further investigation.

In humans, a recent Phase I trial showed that oral baicalein at 100–2800 mg daily (it was equal to about 1.7–46.7 mg/kg per day, standard weight of human was set as 60 kg) displays favorable pharmacokinetic profiles, and the doses are safe and well tolerated [72]. Similarly, in another human clinical trial, oral baicalein at 0, 400, and 800 mg (it was equal to about 6.7 and 13.3 mg/kg, respectively) (once daily at days 1 and 10 and twice daily on days 3–9) for 10 days in healthy Chinese volunteers showed good safety and tolerability [73]. Therefore, baicalein at 100 mg/kg/day (it was equal to about 10 mg/kg/day in human according to conversion of human and mouse body surface) at the present study may be suitable to use in humans as a hepatoprotective agent against acute liver injury. Further clinical trials are still required.

## 5. Conclusions

In conclusion, our results reveal that CCl_4_-induced acute liver injury involved multiple cellular process, including apoptosis, oxidative stress, inflammation, and ferroptosis. Baicalein supplementation ameliorates CCl_4_-induced acute liver injury in mice by upregulating the antioxidant defense functions and downregulating oxidative stress, apoptosis, inflammation and ferrroptosis, which may involve the activation of Nrf2 pathway and the inhibition of ALOX12 and NF-κB pathways. The current study highlights the potential clinical utility of baicalein supplementation for patients suffering from acute liver disease and paves the way for future clinical trials.

## Data Availability

All data in the present study are available on request from corresponding author.

## Supplementary Materials

The following are available online at https://www.mdpi.com/article/10.3390/antiox10060976/s1, Figure S1: Gene expression involved apoptosis, inflammation and ferroptosis pathway in the liver tissues exposed with CCl_4_. Figure S2: Gene and protein expression was detected in HepG2 cells with the knockdown of *ALOX12* and *Nrf2* genes. Figure S3: Effect of baicalein on erastin and CCl_4_-induced cell death in HepG2 cells. Figure S4: Effect of knockdown of *ALOX12* and *Nrf2* on CCl_4_-induced cell death in HepG2 cells. Table S1: Primers for qRT-PCR.

## References

[1] Xiao J., Wang F., Wong N.K., He J., Zhang R., Sun R., Xu Y., Liu Y., Li W., Koike K. (2019). Global liver disease burdens and research trends: Analysis from a chinese perspective. J. Hepatol..

[2] Singh D., Cho W.C., Upadhyay G. (2015). Drug-induced liver toxicity and prevention by herbal antioxidants: An overview. Front. Physiol..

[3] Wu Z., Han M., Chen T., Yan W., Ning Q. (2010). Acute liver failure: Mechanisms of immune-mediated liver injury. Liver Int..

[4] Niu L., Cui X., Qi Y., Xie D., Wu Q., Chen X., Ge J., Liu Z. (2016). Involvement of tgf-beta1/smad3 signaling in carbon tetrachloride-induced acute liver injury in mice. PLoS ONE.

[5] Cong M., Zhao W., Liu T., Wang P., Fan X., Zhai Q., Bao X., Zhang D., You H., Kisseleva T. (2017). Protective effect of human serum amyloid p on ccl4-induced acute liver injury in mice. Int. J. Mol. Med..

[6] Zhang D.G., Zhang C., Wang J.X., Wang B.W., Wang H., Zhang Z.H., Chen Y.H., Lu Y., Tao L., Wang J.Q. (2017). Obeticholic acid protects against carbon tetrachloride-induced acute liver injury and inflammation. Toxicol. Appl. Pharmacol..

[7] Xiao J., Liong E.C., Huang H., On Tse W., Lau K.S., Pan J., Nanji A.A., Fung M.L., Xing F., Tipoe G.L. (2015). Cyclooxygenase-1 serves a vital hepato-protective function in chemically induced acute liver injury. Toxicol. Sci..

[8] Lee J., Giordano S., Zhang J. (2012). Autophagy, mitochondria and oxidative stress: Cross-talk and redox signalling. Biochem. J..

[9] Ding J., Cui X., Liu Q. (2017). Emerging role of hmgb1 in lung diseases: Friend or foe. J. Cell. Mol. Med..

[10] Zou Y., Xiong J.B., Ma K., Wang A.Z., Qian K.J. (2017). Rac2 deficiency attenuates ccl4-induced liver injury through suppressing inflammation and oxidative stress. Biomed. Pharmacother..

[11] Zeng B., Su M., Chen Q., Chang Q., Wang W., Li H. (2017). Protective effect of a polysaccharide from anoectochilus roxburghii against carbon tetrachloride-induced acute liver injury in mice. J. Ethnopharmacol..

[12] Torres L.R., Santana F.C., Torres-Leal F.L., Melo I.L., Yoshime L.T., Matos-Neto E.M., Seelaender M.C., Araujo C.M., Cogliati B., Mancini-Filho J. (2016). Pequi (caryocar brasiliense camb.) almond oil attenuates carbon tetrachloride-induced acute hepatic injury in rats: Antioxidant and anti-inflammatory effects. Food Chem. Toxicol..

[13] Wang M., Niu J., Ou L., Deng B., Wang Y., Li S. (2019). Zerumbone protects against carbon tetrachloride (ccl4)-induced acute liver injury in mice via inhibiting oxidative stress and the inflammatory response: Involving the tlr4/nf-kappab/cox-2 pathway. Molecules.

[14] Peng X., Dai C., Liu Q., Li J., Qiu J. (2018). Curcumin attenuates on carbon tetrachloride-induced acute liver injury in mice via modulation of the nrf2/ho-1 and tgf-beta1/smad3 pathway. Molecules.

[15] Dai C., Xiao X., Li D., Tun S., Wang Y., Velkov T., Tang S. (2018). Chloroquine ameliorates carbon tetrachloride-induced acute liver injury in mice via the concomitant inhibition of inflammation and induction of apoptosis. Cell Death Dis..

[16] Domitrovic R., Jakovac H., Blagojevic G. (2011). Hepatoprotective activity of berberine is mediated by inhibition of tnf-alpha, cox-2, and inos expression in ccl(4)-intoxicated mice. Toxicology.

[17] Wang W., Wang S., Liu J., Cai E., Zhu H., He Z., Gao Y., Li P., Zhao Y. (2018). Sesquiterpenoids from the root of panax ginseng protect ccl4-induced acute liver injury by anti-inflammatory and anti-oxidative capabilities in mice. Biomed. Pharmacother..

[18] Stockwell B.R., Friedmann Angeli J.P., Bayir H., Bush A.I., Conrad M., Dixon S.J., Fulda S., Gascon S., Hatzios S.K., Kagan V.E. (2017). Ferroptosis: A regulated cell death nexus linking metabolism, redox biology, and disease. Cell.

[19] Leu J.I., Murphy M.E., George D.L. (2019). Mechanistic basis for impaired ferroptosis in cells expressing the african-centric s47 variant of p53. Proc. Natl. Acad. Sci. USA.

[20] Cai Z., Lou Q., Wang F., Li E., Sun J., Fang H., Xi J., Ju L. (2015). N-acetylcysteine protects against liver injure induced by carbon tetrachloride via activation of the nrf2/ho-1 pathway. Int. J. Clin. Exp. Pathol..

[21] Mohammed A., Abd Al Haleem E.N., El-Bakly W.M., El-Demerdash E. (2016). Deferoxamine alleviates liver fibrosis induced by ccl4 in rats. Clin. Exp. Pharmacol. Physiol..

[22] Liang W., Huang X., Chen W. (2017). The effects of baicalin and baicalein on cerebral ischemia: A review. Aging Dis..

[23] Sahu B.D., Kumar J.M., Sistla R. (2015). Baicalein, a bioflavonoid, prevents cisplatin-induced acute kidney injury by up-regulating antioxidant defenses and down-regulating the mapks and nf-kappa b pathways. PLoS ONE.

[24] de Oliveira M.R., Nabavi S.F., Habtemariam S., Orhan I.E., Daglia M., Nabavi S.M. (2015). The effects of baicalein and baicalin on mitochondrial function and dynamics: A review. Pharmacol. Res..

[25] Zhang Z., Cui W., Li G., Yuan S., Xu D., Hoi M.P., Lin Z., Dou J., Han Y., Lee S.M. (2012). Baicalein protects against 6-ohda-induced neurotoxicity through activation of keap1/nrf2/ho-1 and involving pkcalpha and pi3k/akt signaling pathways. J. Agric. Food Chem..

[26] Lai C.C., Huang P.H., Yang A.H., Chiang S.C., Tang C.Y., Tseng K.W., Huang C.H. (2016). Baicalein, a component of scutellaria baicalensis, attenuates kidney injury induced by myocardial ischemia and reperfusion. Planta Med..

[27] Xiao T., Cui Y., Ji H., Yan L., Pei D., Qu S. (2021). Baicalein attenuates acute liver injury by blocking nlrp3 inflammasome. Biochem. Biophys. Res. Commun..

[28] Dai C., Tang S., Wang Y., Velkov T., Xiao X. (2017). Baicalein acts as a nephroprotectant that ameliorates colistin-induced nephrotoxicity by activating the antioxidant defence mechanism of the kidneys and down-regulating the inflammatory response. J. Antimicrob. Chemother..

[29] Zhou H.C., Wang H., Shi K., Li J.M., Zong Y., Du R. (2018). Hepatoprotective effect of baicalein against acetaminophen-induced acute liver injury in mice. Molecules.

[30] Dixon S.J., Lemberg K.M., Lamprecht M.R., Skouta R., Zaitsev E.M., Gleason C.E., Patel D.N., Bauer A.J., Cantley A.M., Yang W.S. (2012). Ferroptosis: An iron-dependent form of nonapoptotic cell death. Cell.

[31] Yamada N., Karasawa T., Kimura H., Watanabe S., Komada T., Kamata R., Sampilvanjil A., Ito J., Nakagawa K., Kuwata H. (2020). Ferroptosis driven by radical oxidation of n-6 polyunsaturated fatty acids mediates acetaminophen-induced acute liver failure. Cell Death Dis..

[32] Popović D., Kocić G., Katić V., Zarubica A., Veličković L.J., Ničković V.P., Jović A., Veljković A., Petrović V., Rakić V. (2019). Anthocyanins protect hepatocytes against ccl(4)-induced acute liver injury in rats by inhibiting pro-inflammatory mediators, polyamine catabolism, lipocalin-2, and excessive proliferation of kupffer cells. Antioxidants.

[33] Stine J.G., Intagliata N., Shah N.L., Argo C.K., Caldwell S.H., Lewis J.H., Northup P.G. (2015). Hepatic decompensation likely attributable to simeprevir in patients with advanced cirrhosis. Dig. Dis. Sci..

[34] Cragg G.M., Newman D.J. (2013). Natural products: A continuing source of novel drug leads. Biochim. Biophys. Acta.

[35] Shi J., Aisaki K., Ikawa Y., Wake K. (1998). Evidence of hepatocyte apoptosis in rat liver after the administration of carbon tetrachloride. Am. J. Pathol..

[36] Wu S.J., Lin Y.H., Chu C.C., Tsai Y.H., Chao J.C. (2008). Curcumin or saikosaponin a improves hepatic antioxidant capacity and protects against ccl4-induced liver injury in rats. J. Med. Food.

[37] Dai C., Li J., Tang S., Li J., Xiao X. (2014). Colistin-induced nephrotoxicity in mice involves the mitochondrial, death receptor, and endoplasmic reticulum pathways. Antimicrob. Agents Chemother..

[38] Chen C., Cai C., Lin H., Zhang W., Peng Y., Wu K. (2018). Baicalein protects renal tubular epithelial cells againsthypoxia-reoxygenation injury. Ren. Fail..

[39] Sahu B.D., Kumar J.M., Kuncha M., Borkar R.M., Srinivas R., Sistla R. (2016). Baicalein alleviates doxorubicin-induced cardiotoxicity via suppression of myocardial oxidative stress and apoptosis in mice. Life Sci..

[40] Perez C.A., Wei Y., Guo M. (2009). Iron-binding and anti-fenton properties of baicalein and baicalin. J. Inorg. Biochem..

[41] Dai C., Li B., Zhou Y., Li D., Zhang S., Li H., Xiao X., Tang S. (2016). Curcumin attenuates quinocetone induced apoptosis and inflammation via the opposite modulation of nrf2/ho-1 and nf-kb pathway in human hepatocyte l02 cells. Food Chem. Toxicol..

[42] Wu Y.L., Lian L.H., Wan Y., Nan J.X. (2010). Baicalein inhibits nuclear factor-kappab and apoptosis via c-flip and mapk in d-galn/lps induced acute liver failure in murine models. Chem. Biol. Interact..

[43] Ahmad R., Rasheed Z., Ahsan H. (2009). Biochemical and cellular toxicology of peroxynitrite: Implications in cell death and autoimmune phenomenon. Immunopharmacol. Immunotoxicol..

[44] Choi E.O., Jeong J.W., Park C., Hong S.H., Kim G.Y., Hwang H.J., Cho E.J., Choi Y.H. (2016). Baicalein protects c6 glial cells against hydrogen peroxide-induced oxidative stress and apoptosis through regulation of the nrf2 signaling pathway. Int. J. Mol. Med..

[45] Dai C., Tang S., Deng S., Zhang S., Zhou Y., Velkov T., Li J., Xiao X. (2015). Lycopene attenuates colistin-induced nephrotoxicity in mice via activation of the nrf2/ho-1 pathway. Antimicrob. Agents Chemother..

[46] Dai C., Chen X., Li J., Comish P., Kang R., Tang D. (2020). Transcription factors in ferroptotic cell death. Cancer Gene Ther..

[47] Yeh C.H., Ma K.H., Liu P.S., Kuo J.K., Chueh S.H. (2015). Baicalein decreases hydrogen peroxide-induced damage to ng108-15 cells via upregulation of nrf2. J. Cell Physiol..

[48] Shi L., Hao Z., Zhang S., Wei M., Lu B., Wang Z., Ji L. (2018). Baicalein and baicalin alleviate acetaminophen-induced liver injury by activating nrf2 antioxidative pathway: The involvement of erk1/2 and pkc. Biochem. Pharmacol..

[49] Jeong J.Y., Cha H.J., Choi E.O., Kim C.H., Kim G.Y., Yoo Y.H., Hwang H.J., Park H.T., Yoon H.M., Choi Y.H. (2019). Activation of the nrf2/ho-1 signaling pathway contributes to the protective effects of baicalein against oxidative stress-induced DNA damage and apoptosis in hei193 schwann cells. Int. J. Med. Sci..

[50] Sun X., Ou Z., Chen R., Niu X., Chen D., Kang R., Tang D. (2016). Activation of the p62-keap1-nrf2 pathway protects against ferroptosis in hepatocellular carcinoma cells. Hepatology.

[51] Dai C., Zhang D., Li J., Li J. (2013). Effect of colistin exposure on calcium homeostasis and mitochondria functions in chick cortex neurons. Toxicol. Mech. Methods.

[52] Jang S., Yu L.R., Abdelmegeed M.A., Gao Y., Banerjee A., Song B.J. (2015). Critical role of c-jun n-terminal protein kinase in promoting mitochondrial dysfunction and acute liver injury. Redox Biol..

[53] Yu Z., Li Q., Wang Y., Li P. (2020). A potent protective effect of baicalein on liver injury by regulating mitochondria-related apoptosis. Apoptosis.

[54] Choi Y.H. (2019). Activation of the nrf2/ho-1 signaling pathway contributes to the protective effects of coptisine against oxidative stress-induced DNA damage and apoptosis in hacat keratinocytes. Gen. Physiol. Biophys..

[55] Zhang S., Lu B., Han X., Xu L., Qi Y., Yin L., Xu Y., Zhao Y., Liu K., Peng J. (2013). Protection of the flavonoid fraction from rosa laevigata michx fruit against carbon tetrachloride-induced acute liver injury in mice. Food Chem. Toxicol..

[56] Shi H., Han W., Shi H., Ren F., Chen D., Chen Y., Duan Z. (2017). Augmenter of liver regeneration protects against carbon tetrachloride-induced liver injury by promoting autophagy in mice. Oncotarget.

[57] Cao M., Wang H., Guo L., Yang S., Liu C., Khor T.O., Yu S., Kong A.N. (2017). Dibenzoylmethane protects against ccl4-induced acute liver injury by activating nrf2 via jnk, ampk, and calcium signaling. AAPS J..

[58] Yang B.Y., Zhang X.Y., Guan S.W., Hua Z.C. (2015). Protective effect of procyanidin b2 against ccl4-induced acute liver injury in mice. Molecules.

[59] Morgan M.J., Liu Z.G. (2011). Crosstalk of reactive oxygen species and nf-kappab signaling. Cell Res..

[60] Xiang L., Hu Y.F., Wu J.S., Wang L., Huang W.G., Xu C.S., Meng X.L., Wang P. (2018). Semi-mechanism-based pharmacodynamic model for the anti-inflammatory effect of baicalein in lps-stimulated raw264.7 macrophages. Front. Pharmacol..

[61] Sun H., Che Q.M., Zhao X., Pu X.P. (2010). Antifibrotic effects of chronic baicalein administration in a ccl4 liver fibrosis model in rats. Eur. J. Pharmacol..

[62] Huang H.L., Wang Y.J., Zhang Q.Y., Liu B., Wang F.Y., Li J.J., Zhu R.Z. (2012). Hepatoprotective effects of baicalein against ccl(4)-induced acute liver injury in mice. World J. Gastroenterol..

[63] Zahedi K., Barone S.L., Xu J., Steinbergs N., Schuster R., Lentsch A.B., Amlal H., Wang J., Casero R.A., Soleimani M. (2012). Hepatocyte-specific ablation of spermine/spermidine-n1-acetyltransferase gene reduces the severity of ccl4-induced acute liver injury. Am. J. Physiol. Gastrointest. Liver Physiol..

[64] Huang G.J., Deng J.S., Chiu C.S., Liao J.C., Hsieh W.T., Sheu M.J., Wu C.H. (2012). Hispolon protects against acute liver damage in the rat by inhibiting lipid peroxidation, proinflammatory cytokine, and oxidative stress and downregulating the expressions of inos, cox-2, and mmp-9. Evid. Based Complement. Alternat. Med..

[65] Xie Y., Song X., Sun X., Huang J., Zhong M., Lotze M.T., Zeh H.J.R., Kang R., Tang D. (2016). Identification of baicalein as a ferroptosis inhibitor by natural product library screening. Biochem. Biophys. Res. Commun..

[66] Yang W.S., SriRamaratnam R., Welsch M.E., Shimada K., Skouta R., Viswanathan V.S., Cheah J.H., Clemons P.A., Shamji A.F., Clish C.B. (2014). Regulation of ferroptotic cancer cell death by gpx4. Cell.

[67] Fang X., Wang H., Han D., Xie E., Yang X., Wei J., Gu S., Gao F., Zhu N., Yin X. (2019). Ferroptosis as a target for protection against cardiomyopathy. Proc. Natl. Acad. Sci. USA.

[68] Li Q., Li Q.Q., Jia J.N., Sun Q.Y., Zhou H.H., Jin W.L., Mao X.Y. (2019). Baicalein exerts neuroprotective effects in fecl3-induced posttraumatic epileptic seizures via suppressing ferroptosis. Front. Pharmacol..

[69] Tesfay L., Paul B.T., Konstorum A., Deng Z., Cox A.O., Lee J., Furdui C.M., Hegde P., Torti F.M., Torti S.V. (2019). Stearoyl-coa desaturase 1 protects ovarian cancer cells from ferroptotic cell death. Cancer Res..

[70] Lu M.J., Chen Y.S., Huang H.S., Ma M.C. (2014). Hypoxic preconditioning protects rat hearts against ischemia-reperfusion injury via the arachidonate12-lipoxygenase/transient receptor potential vanilloid 1 pathway. Basic Res. Cardiol..

[71] Xue Y., Deng Q., Zhang Q., Ma Z., Chen B., Yu X., Peng H., Yao S., Liu J., Ye Y. (2020). Gigantol ameliorates ccl4-induced liver injury via preventing activation of jnk/cpla2/12-lox inflammatory pathway. Sci. Rep..

[72] Li M., Shi A., Pang H., Xue W., Li Y., Cao G., Yan B., Dong F., Li K., Xiao W. (2014). Safety, tolerability, and pharmacokinetics of a single ascending dose of baicalein chewable tablets in healthy subjects. J. Ethnopharmacol..

[73] Pang H., Xue W., Shi A., Li M., Li Y., Cao G., Yan B., Dong F., Xiao W., He G. (2016). Multiple-ascending-dose pharmacokinetics and safety evaluation of baicalein chewable tablets in healthy chinese volunteers. Clin. Drug Investig..

