# Supplementary material for: Inhibition of Oxidative Stress and ALOX12 and NF-κB Pathways Contribute to the Protective Effect of Baicalein on Carbon Tetrachloride-Induced Acute Liver Injury
Source: Antioxidants (Basel). 2021 Jun 18;10(6):976. doi: 10.3390/antiox10060976 (PMC8235740; doi:10.3390/antiox10060976)
Supplement: Supplementary file 1 [file antioxidants-10-00976-s001.zip › antioxidants-1215182-supplementary.pdf]

Supplemental data

Inhibition of oxidative stress and ALOX12 and NF-kB pathways contribute to the protective effect of baicalein on carbon tetrachloride-induced acute liver injury

Chongshan Dai [1,2*], Hui Li[3], Yang Wang [1,2], Shusheng Tang [1,2], Tony Velkov [4*], Jianzhong Shen[1, 2]

[1]College of Veterinary Medicine, China Agricultural University, No. 2 Yuanmingyuan West Road, Beijing 100193, P. R. China.

[2]Beijing Key Laboratory of Detection Technology for Animal-Derived Food Safety, College of Veterinary Medicine, China Agricultural University, Beijing 100193, P. R. China.

[3]University of Texas Southwestern Medical Center, Dallas, Texas, USA.

[4]Department of Pharmacology & Therapeutics, School of Biomedical Sciences, Faculty of Medicine, Dentistry and Health Sciences, The University of Melbourne, Parkville, Victoria, 3010, Australia.

*Corresponding authors:

Chongshan Dai, E-mail: daichongshan@cau.edu.cn; OR Tony Velkov, Tony.Velkov@unimelb.edu.au

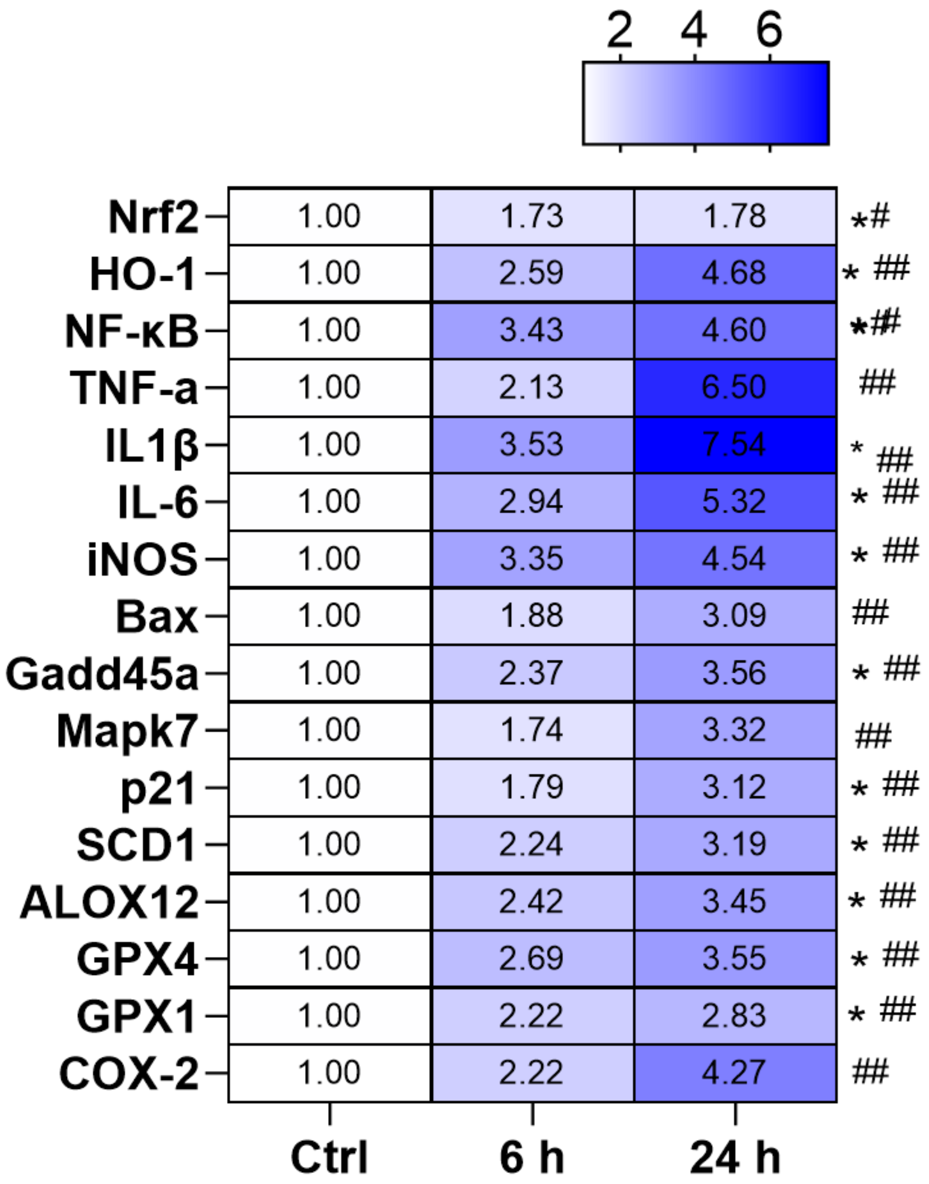

Figure S1. Gene expression involved apoptosis, inflammation and ferroptosis pathway in the liver tissues exposed with CCl_4. Heat map of gene expression in the liver tissues of mice exposed to CCl4 at 6 and 24 h (n = 4). 6 h vs ctrl, * $p < 0.05$, ** $p < 0.01$; 24 h vs ctrl, # $p < 0.05$, ## $p < 0.01$. Ctrl, control.

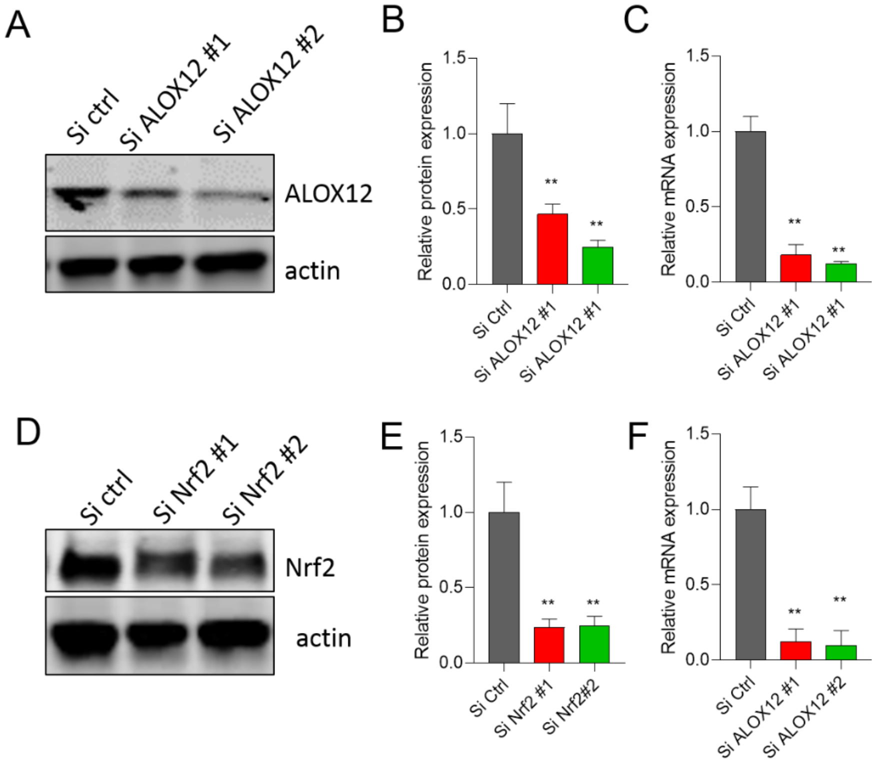

Figure S2. Gene and protein expression was detected in HepG2 cells with the knockdown of *ALOX12* and *Nrf2* genes. (A) Representative expression of ALOX12 protein by western blot after HepG2 cells were transfected with SiRNA#1 (SASI_Hs02_00303100) and SiRNA#2, (SASI_Hs02_00303101), respectively. (B) and (C) Levels of protein and mRNA in HepG2 cells after SiRNA transfection were determined. (D) Representative expression of Nrf2 protein by western blot after HepG2 were transfected with SiRNA#1 (SASI_Hs01_00182393) and SiRNA#2 (SASI_Hs02_00341015), respectively. The protein quantification and mRNA expression showed in (E) and (F), respectively. Data are showed as mean ± SD. *$p < 0.05$, **$p < 0.01$, compared to the Si Ctrl group, respectively. Ctrl, control.

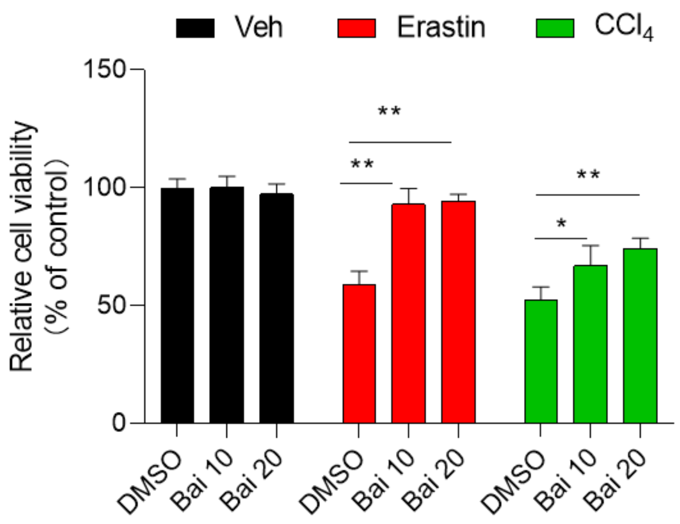

Figure S3. Effect of baicalein on erastin and CCl_4-induced cell death in HepG2 cells. HepG2 cells were treated with baicalein at the doses of 10 or 20 μM for 2 h, then cells were treated with 10 μM erastin or 0.4% CCl_4 for additional 24 h. Cell viabilities were finally examined. Data are presented as mean ± SD (n=5). $^*p < 0.05$, $^{**}p < 0.01$.

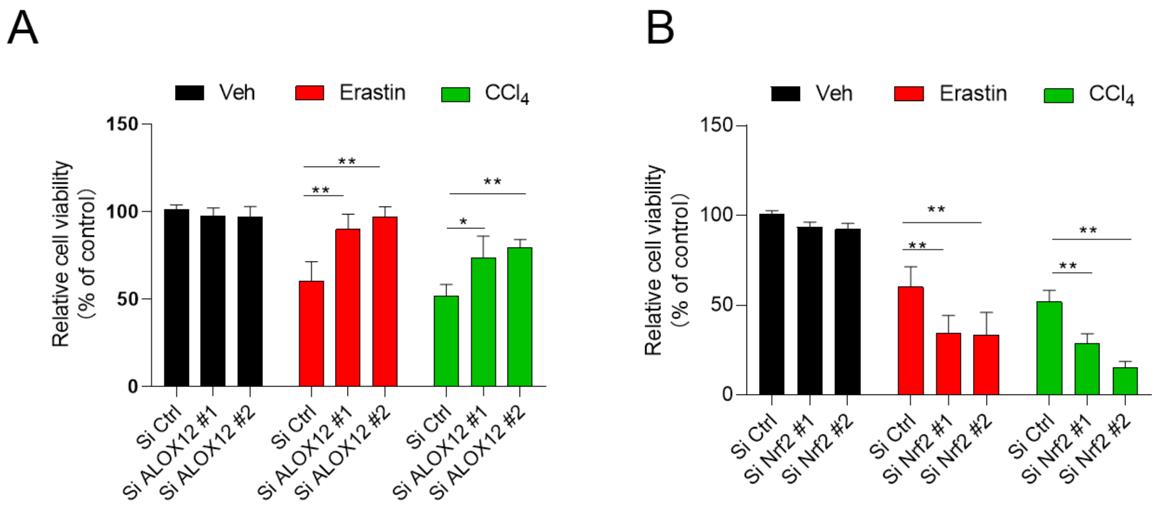

Figure S4. Effect of knockdown of ALOX12 and Nrf2 on CCl_4 and erastin-induced cell death in HepG2 cells. (A) Effects of knockdown of ALOX12 (A) or Nrf2 (B) by SiRNA on erastin (10 μM) or CCl_4 (0.4%) treatment (for 24 h) -induced the loss of cell viabilities. All data are presented as mean ± SD (n = 3). *p < 0.05, **p < 0.01.

Table S1. Primers for qRT-PCR

β-Actin	Mouse	5′-GCCCTGAGGCTCTTTTCCA-3′
		5′-GTTGGCATAGAGGTCTTTACGGAT-3′
Gadd45a	Mouse	5′-GCAGAGCAGAAGACCGAAAG-3′
		5′-TAACAGAACGCACGGATGAG-3′
Mapk7	Mouse	5′- TGTGACCAATGCCAAACGG-3′
		5′-GCGGCTGTGAAGAGTGAATGA-3′
Nrf2	Mouse	5′-CAC ATT CCC AAA CAA GAT GC-3′
		5′-TCT TTT TCC AGC GAG GAG AT-3′
HO-1	Mouse	5′-CGT GCT CGA ATG AAC ACT CT-3
		5′-GGA AGC TGA GAG TGA GGA CC-3′;
NF-κB	Mouse	5′-CAC TGT CTG CCT CTC TCG TCT-3′
		5′-AAG GAT GTC TCC ACA CCA CTG-3′;
COX-2	Mouse	5′-CAA GCA GTG GCA AAG GCC TCC A-3′
		5′-GGC ACT TGC ATT GAT GGT GGC T-3
Alox12	Mouse	5′-TCCCTCAACCTAGTGCGTTTG-3′
		5′-GTTGCAGCTCCAGTTTCGC-3′
TNF	Mouse	5′-AGCCGATGGGTTGTACCTTG-3′
		5′-ATAGCAAATCGGCTGACGGT-3′
IL1B	Mouse	5′-CCGTGGACCTTCCAGGATGA-3′
		5′-GGGAACGTCACACACCAGCA-3′
IL-6	Mouse	5′-AGGATACCACTCCCAACAGACCT-3′

		5′-CAAGTGCATCATCGTTGTTCATAC-3′
iNOS	Mouse	5′-GGCAGCCTGTGAGACCTTTG-3′
		5′-GCATTGGAAGTGAAGCGTTTC-3′
Bax	Mouse	5′-AAACTGGTGCTCAAGGCCCT-3′
		5′-AGCAGCCGCTCACGGAG-3′
GPX4	Mouse	5′-TTCCCGTGTAACCAGTTCG-3′
		5′-CGGCGAACTCTTTGATCTCT-3′
GPX1	Mouse	5′-TCGGTTTCCCGTGCAATCAG-3′
		5′-GTCGGACGTACTTGAGGGAA-3′
SCD1	Mouse	5′-GCAAGCTCTACACCTGCCTCTT-3′
		5′-CGTGCCTTGTAAGTTCTGTGGC-3′
Alox12	Human	5′-CCTCGTTATGCTGAAGATGGAGC-3′
		5′-ATTTCGGACCCAGGACTTTGCC-3′
β-Actin	Human	5′-CACCATTGGCAATGAGCGGTTC-3′
		5′-AGGTCTTTGCGGATGTCCACGT-3′
Nrf2	Human	5′-CACATCCAGTCAGAAACCAGTGG-3′
		5′-GGAATGTCTGCGCCAAAAGCTG-3′
HO-1	Human	5′-CCAGGCAGAGAATGCTGAGTTC-3′
		5′-AAGACTGGGCTCTCCTTGTTGC-3′
